# Research progress in flap temperature monitoring technologies and applications after transplantation

**DOI:** 10.1063/5.0314573

**Published:** 2026-04-09

**Authors:** Tingting Hu, Tao Yan, Junjie Chen, Qin Huang, Miao Cui, Yanting Han, Ka Li

**Affiliations:** 1 Sichuan University-The Hong Kong Polytechnic University, Institute for Disaster Management and Reconstruction, Chengdu 610041, China; 2 Medicine and Engineering Interdisciplinary Research Laboratory of Nursing & Materials, West China Hospital, Sichuan University/West China School of Nursing, Sichuan University, Chengdu 610041, China; 3 Deyang People's Hospital, Deyang 618000, China; 4 Stanford Cardiovascular Institute, Stanford University, Stanford, California 94305, USA; 5 Department of Genetics, Stanford University School of Medicine, Stanford, California 94305, USA; 6 Tianfu Jincheng Laboratory, Chengdu 610093, China

## Abstract

As a primary method for tissue repair and functional reconstruction, flap transplantation has achieved a clinical success rate of over 90%. However, the postoperative incidence of vascular crisis remains as high as 10%–30%, making it a leading cause of reoperation and disability. Flap temperature is a key indicator closely correlated with microcirculatory status, making its monitoring essential for the early detection of complications. This paper provides a systematic review of the physiological mechanisms underlying postoperative flap temperature, the characteristic temperature changes associated with venous and arterial crises, and the latest advancements in monitoring technologies. It comprehensively analyzes the principles, advantages, and limitations of various methods, including manual palpation, contact thermometry, infrared thermography, fiber optic sensing, and microwave thermometry. Furthermore, the review explores the application of intelligent technologies such as wearable sensors, artificial intelligence-driven predictive systems, implantable flexible devices, and multimodal fusion monitoring. Current challenges, including poor real-time performance, low precision, and a lack of standardization, are highlighted. Future development is directed toward precision, intelligence, and integration, with an emphasis on multidisciplinary collaboration to create more accurate, convenient, and intelligent monitoring systems. These advancements aim to achieve precise early warning and timely intervention, ultimately improving flap survival rates and patient outcomes.

## I. INTRODUCTION

Flap transplantation is a tissue transfer technique that transplants skin and subcutaneous tissue with its intrinsic blood supply from the donor site to the recipient site. As a primary therapeutic approach for tissue repair and organ function reconstruction, it achieves the synergistic effects of wound revascularization, anti-infective barrier establishment, and functional restoration. Characterized by a broad repair scope and effective improvement of the local microenvironment, this technique has become the “gold standard” for the treatment of numerous refractory wounds.^1–3^ Relevant data indicate that in China, the annual volume of flap transplantation surgeries exceeds 150 000 cases. With the popularization of high-precision transplantation technologies such as perforator flaps and free flaps, the surgical indications have expanded from traditional trauma management to complex scenarios, including congenital malformations and chronic non-healing wounds.^4,5^ In recent years, the clinical success rate of flap transplantation has increased to over 90%. However, literature reports show that the incidence of postoperative vascular crisis ranges from 10% to 30%, leading to a secondary surgery rate of 25% and a disability rate of 13.5%, which poses a significant threat to patient prognosis.^6,7^

Flap microcirculation refers to the entire microcirculatory system that sustains the survival of transplanted flaps.^8^ Abnormalities in its structure or function will directly induce microcirculatory disturbance, accompanied by complications such as vascular crisis.^4^ The underlying causes include environmental factors (e.g., excessive tightness of dressings, positional compression, etc.)^5^ and physiological factors (e.g., vascular spasm, anastomotic stenosis, etc.).^6^ The onset of flap microcirculatory disturbance exhibits a stepwise progression over time. In the early stage, it is predominantly characterized by vascular spasm or microthrombosis, with insidious clinical manifestations that are prone to misdiagnosis. If not detected promptly, it progresses to venous congestion within hours, ultimately leading to tissue ischemia and necrosis that requires secondary surgery.^9^ However, the onset of microcirculatory disturbance is not irreversible; early accurate identification and intervention can prevent deterioration, thereby ensuring flap survival by improving and reconstructing microcirculation.^10^ In the nursing practice of flap transplantation, nurses' observation and evaluation of flap status, followed by timely feedback to the medical team, constitute a crucial link in blocking the progression of microcirculatory disturbance.^11,12^ Systematic reviews of existing literature have revealed that flap temperature is closely associated with microcirculatory disturbance and possesses monitorable and identifiable characteristics. This implies that nurses can achieve early warning of flap microcirculatory disturbance through dynamic monitoring of changes in physiological indicators such as flap temperature.

Clinical methods for monitoring flap temperature mainly include traditional finger palpation, infrared thermometer measurement, and infrared thermographic camera measurement.^13^ Among these, finger palpation relies on the subjective perception of medical staff, infrared thermometers focus on local single-point temperature collection, and infrared thermographic cameras enable visualization of the overall temperature distribution. However, these methods still exhibit numerous limitations in practical applications:^14,15^ (1) Insufficient continuity and timeliness, failing to achieve bedside continuous monitoring; (2) Low accuracy and stability, as examination results are susceptible to interference from patient posture and the environment; (3) Weak research on dynamic laws and quantitative correlations, as they only record single-dimensional data and lack multimodal integrated analysis. (4) Currently, the clinical approaches for flap temperature monitoring share common problems, including poor real-time performance, low accuracy, single-dimensional indicators, and low intelligence level.^16,17^

**FIG. 1.**
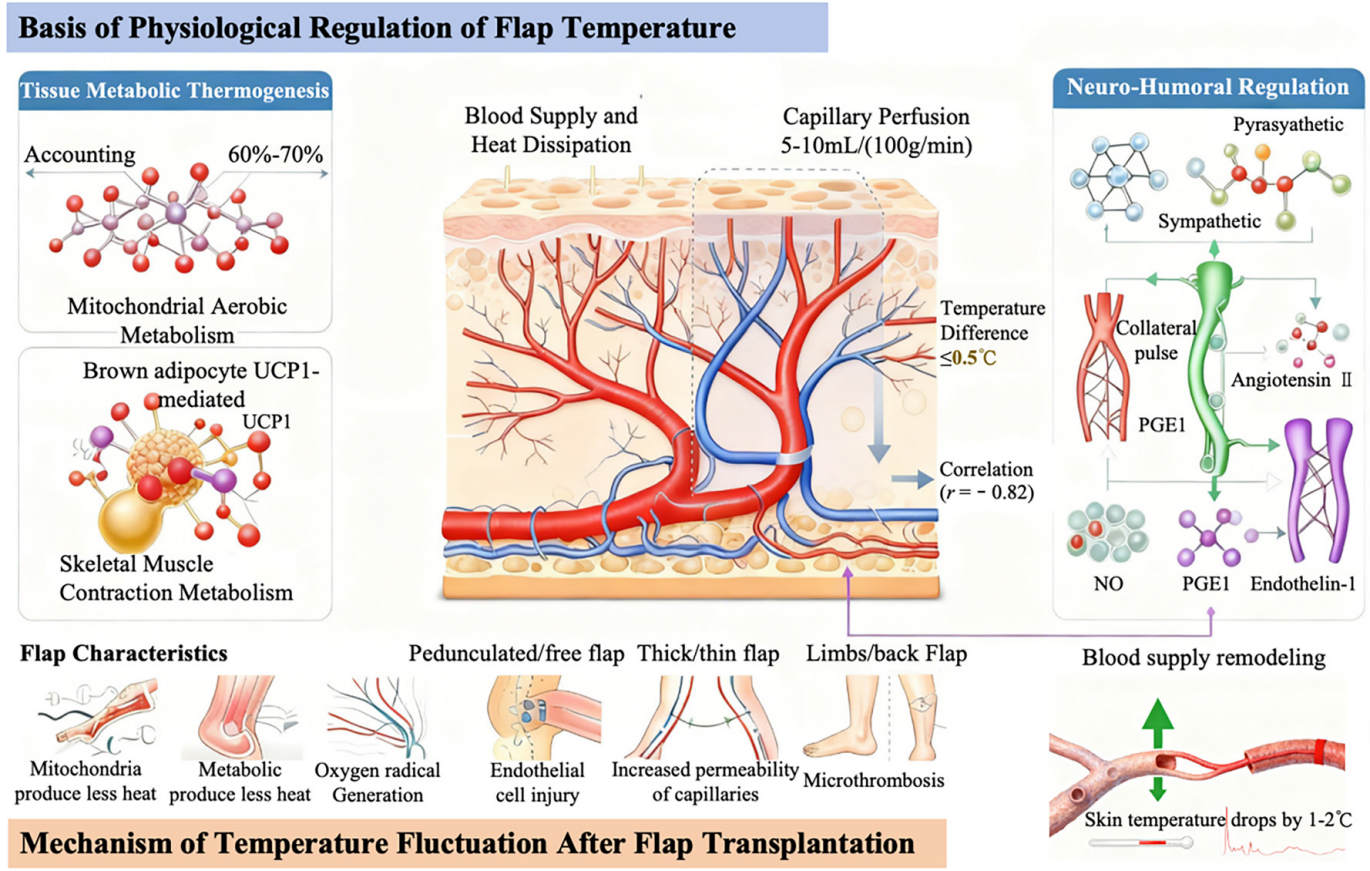
Fundamentals of physiological regulation of flap temperature.

Therefore, based on the interdisciplinary perspective of biomedical engineering, this study conducts a systematic review through a three-dimensional framework of “mechanism-technology-application”: First, starting from the pathophysiological and biophysical mechanisms of microcirculation reconstruction, it reveals the correlation laws between microangiogenesis, hemodynamic remodeling, and temperature changes after flap transplantation and clarifies the biophysical basis of temperature fluctuations at different repair stages; second, based on the classification of biomedical engineering technologies, it systematically elaborates on the working principles, key points of engineering design, and clinically applicable scenarios of existing temperature monitoring technologies; finally, it integrates correlative evidence between temperature parameters and complications such as vascular crisis and flap necrosis and refines the temperature early warning thresholds for different flap types through biostatistical and machine learning methods. This work provides theoretical support and clinical practice reference for the development of precision and personalized intelligent temperature-controlled dressings and closed-loop early warning systems (Fig. 1).

## II. PHYSIOLOGICAL MECHANISMS OF TEMPERATURE MONITORING AFTER FLAP TRANSPLANTATION

### A. Physiological basis of flap temperature regulation

The homeostasis maintenance of flap temperature is based on the structural foundation of the cutaneous microcirculatory network, with its core regulatory logic revolving around the dynamic balance of “heat transport—metabolic heat production—heat dissipation regulation.” The cutaneous microcirculation is composed of arterial branches, capillary networks, arteriovenous anastomoses, and venous return systems. Arterial blood carries the core body heat through the capillaries, transmitting heat to flap tissues via heat conduction between blood and interstitial fluid, while venous blood removes excess heat generated by tissue metabolism, forming a “blood supply—heat dissipation” closed loop.^18^ Under normal physiological conditions, the capillary perfusion of the flap is maintained at 5–10 ml/(100 g/min), the temperature difference between the flap and the corresponding contralateral healthy site is ≤0.5 °C, and it exhibits a significant positive correlation with perfusion volume (r = 0.82).^19^

Tissue metabolism constitutes a crucial supplementary source for maintaining flap temperature. Among various metabolic pathways, the heat generated by mitochondrial aerobic metabolism accounts for 60%–70% of the total heat production in flaps. Heat production in adipose tissue is mediated by the UCP1 protein in brown adipocytes, while basal contractile metabolism of skeletal muscle can also generate a small amount of heat.^20^ For thin flaps (thickness < 3 mm) or fasciocutaneous flaps, due to the low proportion of adipose and muscle tissues, the contribution of metabolic heat production is limited. Their temperature is more dependent on heat transport via arterial blood supply, thus rendering them more sensitive to changes in blood perfusion.

Neurohumoral regulation serves as the key regulatory pathway for temperature homeostasis: Sympathetic nerves release norepinephrine to act on α-receptors on vascular smooth muscle, inducing vasoconstriction, reducing blood perfusion, and thereby decreasing skin temperature. In contrast, parasympathetic activation or sympathetic inhibition leads to vasodilation and increased blood flow, resulting in elevated skin temperature.^21^ Among humoral factors, vasodilators such as nitric oxide (NO) and prostaglandin E1 (PGE1) can activate guanylate cyclase in vascular smooth muscle, dilating blood vessels to enhance heat transport, whereas vasoconstrictors including angiotensin II and endothelin-1 reduce heat loss by constricting blood vessels.^22^ Furthermore, intrinsic characteristics of flaps and inherent differences in transplantation environments also affect the temperature baseline: Pedicled flaps, which retain their original blood supply arteries and neural innervation, exhibit superior microcirculatory stability compared to free flaps; thick flaps (with a subcutaneous fat layer > 5 mm) possess a more abundant capillary network, and the thermal insulation effect of adipose tissue can minimize heat loss, resulting in a skin temperature of 0.3–0.6 °C higher than that of thin flaps; extremity flaps, being distant from the core body region and having a large heat dissipation area exposed to the external environment, have a baseline temperature of 0.5–1.0 °C lower than that of trunk and thoracoabdominal flaps.^23^

### B. Correlation between postoperative key physiological changes and temperature fluctuations

After flap transplantation, temperature fluctuations essentially reflect the process of “impairment of physiological regulatory mechanisms—compensatory repair” and are closely associated with blood supply reconstruction, ischemia-reperfusion injury, and systemic-local regulatory imbalance.

In the initial stage of blood flow recanalization (0–6 h) following vascular anastomosis, vascular spasm is prone to occur due to vascular endothelial injury, mechanical stimulation at the anastomotic site, and stress-induced excitation of sympathetic nerve endings. This leads to a decrease in capillary perfusion, resulting in a flap temperature of 1.0–2.0 °C lower than that of the contralateral healthy site.^24^ In most cases, with endothelial repair and degradation of vasoconstrictors, the spasm is relieved, and the perfusion volume recovers to 60%–80% of the normal level, accompanied by a gradual rise in flap temperature—this is defined as physiological temperature fluctuation. However, if the spasm persists for more than 24 h or anastomotic stenosis occurs, the flap temperature remains persistently low, indicating impaired blood supply reconstruction.^25^

Ischemia-reperfusion injury represents the core pathophysiological mechanism underlying abnormal temperature following free flap transplantation:^26^ During the ischemic phase, flap tissues experience mitochondrial dysfunction due to hypoxia, resulting in reduced metabolic heat production and a significant decrease in flap temperature. Following reperfusion, excessive reactive oxygen species (ROS) are generated, which attack vascular endothelial cells, inducing increased capillary permeability and microthrombosis. Even after the restoration of arterial blood supply, local tissues still exhibit no elevation in flap temperature or a fluctuation amplitude >0.5 °C/h due to insufficient perfusion.^27^ Studies have confirmed that when the ischemic time exceeds 6 h, the recovery rate of flap temperature after reperfusion decreases to 0.1–0.2 °C/h, which is positively correlated with the flap necrosis rate (r = 0.78).^28^

## III. RELATIONSHIPS BETWEEN TEMPERATURE AND POSTOPERATIVE COMPLICATIONS OF FLAP TRANSPLANTATION

### A. Venous crisis

Venous crisis is one of the common complications after flap transplantation, primarily caused by venous reflux obstruction. Characteristic changes in flap temperature occur during its occurrence and progression. In the early stage of venous crisis, impaired venous blood return leads to blood stagnation within the flap, resulting in slowed local blood flow and inadequate clearance of metabolic byproducts. This induces a transient elevation in local flap temperature, typically 1–2 °C higher than that of the contralateral healthy skin.^29^ This phase has a relatively short duration, typically several hours. With the progression of the condition, venous congestion aggravates, leading to increasingly prominent hypoxia in flap tissues. Aerobic metabolism is suppressed while anaerobic glycolysis is enhanced, resulting in massive accumulation of acidic metabolic byproducts such as lactic acid. This impairs cellular function and reduces heat production, causing a gradual decrease in flap temperature.^30^ If the venous crisis is not promptly addressed, the flap temperature continues to decline, with the temperature difference from the contralateral healthy skin exceeding 3 °C. Concomitant manifestations include cyanosis of the flap, significant swelling, and prolonged capillary refill time.

Clinical studies have demonstrated that dynamic changes in flap temperature can serve as a crucial indicator for the early identification of venous crisis.^31^ Researchers monitored 120 patients undergoing free flap transplantation and found that 85% of them exhibited flap temperature fluctuations 6–12 h prior to the onset of venous crisis, characterized by a transient initial elevation followed by a gradual decrease. Notably, the rate of temperature decline exhibits a positive correlation with the degree of venous obstruction.^32^ When the flap temperature decreases by more than 0.5 °C per hour, it indicates a high risk of venous crisis progression, requiring prompt intervention.^33^ Furthermore, scholars have pointed out that when the flap temperature is lower than 36.05 °C within 1–3 days postoperatively, it warrants vigilance for the occurrence of vascular crisis.^34^ Integrating flap temperature monitoring with indicators such as the degree of swelling and skin tension can improve the diagnostic accuracy of venous crisis, with a sensitivity exceeding 90%.^35^ Therefore, continuous monitoring of flap temperature changes, particularly focusing on fluctuations within 24–72 h postoperatively, is of great significance for the early detection and management of venous crisis, thereby improving flap prognosis.

### B. Arterial crisis

Arterial crisis is primarily attributed to arterial insufficiency or obstruction, and a rapid, sustained decrease in flap temperature constitutes one of its most prominent characteristics.^36^ Arterial blood flow serves as the primary source of oxygen and nutrients for flap tissues. When vascular spasm, embolism, or torsion of the vascular pedicle occur, the flap instantly loses adequate blood supply, leading to the rapid disruption of aerobic metabolism and a sharp reduction in heat production. Consequently, the flap temperature decreases significantly within a short period.

Clinical studies have demonstrated that within 30 min after the onset of arterial crisis, the flap temperature can decrease by 2–4 °C compared with the contralateral healthy site, with a rapid rate of decline (typically exceeding 1 °C per hour).^37^ Meanwhile, the flap becomes pale in color, accompanied by a markedly prolonged capillary refill time and reduced skin tension—manifestations that exhibit distinct differences from those of venous crisis.

Temperature changes associated with arterial crisis exhibit significant early warning value. Studies have shown that flap temperature is significantly positively correlated with arterial blood flow—when arterial blood flow is reduced by more than 50%, a noticeable decrease in flap temperature occurs.^38^ A retrospective analysis of 95 patients with arterial crisis revealed that all patients experienced a decrease in flap temperature prior to the onset of typical clinical symptoms such as pallor and absent capillary refill, among whom 70% exhibited temperature reduction 2–4 h earlier than other symptoms.^39^ Therefore, adopting “flap temperature persistently 3 °C lower than the contralateral healthy site for more than 1 h without recovery” as the early warning threshold for arterial crisis enables early diagnosis, striving for time for vascular exploration surgery. Furthermore, the recovery rate of flap temperature after the resolution of arterial crisis can reflect the status of blood supply recovery: if the temperature rises by more than 1 °C within 1 h post-intervention, it indicates favorable restoration of arterial blood flow and a high probability of flap survival; conversely, there may be a risk of vascular reocclusion.^40^

**FIG. 2.**
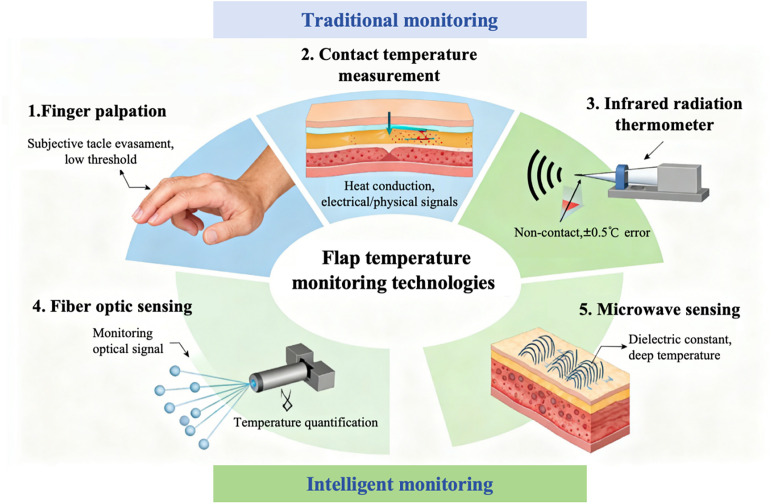
Flap temperature monitoring technologies.

The characteristic temperature changes of arterial crisis provide clinical targets for the research and development of monitoring technologies. At present, flap temperature monitoring technologies applied in clinical practice and scientific research can be divided into the following five categories according to their measurement principles (see in Fig. 2).

## IV. RESEARCH PROGRESS IN FLAP TEMPERATURE MONITORING TECHNOLOGIES

Based on their temperature measurement principles, flap temperature monitoring technologies can be classified into five categories: finger palpation, contact-based temperature measurement technology, infrared radiation temperature measurement technology, fiber optic sensor-based temperature measurement technology, and microwave sensor-based temperature measurement technology. Each type of technology achieves temperature sensing based on distinct physical mechanisms and exhibits unique advantages and limitations in clinical applications, which are summarized as follows.

### A. Finger palpation

Finger palpation involves medical staff touching the flap and the corresponding contralateral healthy skin with the finger pads, relying on subjective tactile perception to judge temperature differences and thereby indirectly evaluate blood supply status. The core advantages of this method lie in its low technical threshold and no requirement for special equipment, enabling rapid preliminary assessment at the bedside at any time. It holds certain practical value especially in primary medical institutions with limited equipment conditions or in emergency rescue scenarios.^41^

However, finger palpation has inherent insurmountable limitations: First and foremost, its accuracy is highly dependent on the operator's clinical experience and tactile sensitivity, leading to significant individual subjective bias and low consistency in temperature judgment of the same flap among different medical staff; second, its ability to identify subtle temperature changes is limited—the perceptual threshold of human finger skin for temperature differences is approximately 1.5–2 °C, making it difficult to detect subclinical temperature fluctuations in early vascular crisis; third, it cannot achieve quantitative recording of temperature values, only allowing qualitative descriptions such as “low skin temperature” or “normal skin temperature,”^42^ which is unfavorable for dynamic trend analysis and objective efficacy assessment. Relevant research data have shown that for early abnormal flap blood supply with a temperature difference < 2 °C, the detection rate of finger palpation is less than 50%, notably lower than that of objective measurement technologies. This results in a high risk of missed diagnosis for this method in the early warning of vascular crisis.^43^

### B. Contact-based temperature measurement technology

The working principle of contact-based temperature measurement technology relies on the heat conduction effect, involving direct contact between the temperature sensor and the flap surface or subcutaneous tissue.^44^ After thermal equilibrium is achieved through heat exchange between the sensor and the measured site, the temperature signal is converted into electrical or physical signals for measurement. A key requirement for this type of technology is the tight adherence between the sensor and flap tissue, ensuring that the heat conduction process is not interfered by external factors such as air gaps, thus guaranteeing measurement accuracy.

In flap monitoring, prominent representatives of contact-based temperature measurement technology include thermocouples and thermistors. Thermocouples consist of a closed circuit formed by two dissimilar metal conductors (e.g., copper-constantan, nickel–chromium–nickel–silicon). When a temperature difference exists between the two junctions, a thermoelectric electromotive force is induced in the circuit, and temperature quantification is achieved based on the linear relationship between electromotive force and temperature. Characterized by rapid response speed and a wide measurement range, thermocouples were extensively applied in real-time postoperative temperature monitoring and intraoperative blood supply assessment of flaps in early clinical practice.^45^ On the other hand, thermistors rely on the exponential resistance-temperature dependence of semiconductor materials (e.g., metal oxides). They exhibit high sensitivity but are strongly susceptible to ambient temperature fluctuations; thus, in clinical settings, they must be used in conjunction with constant-temperature sleeves or thermal shielding devices to eliminate environmental interference. In recent years, the advancement of flexible electronics technology has driven the innovation of contact-based sensors. For instance, wearable flexible thin-film sensors utilize ultra-thin substrates of polyimide (PI) or polydimethylsiloxane (PDMS), achieving conformal adhesion to the flap surface through micro-nano fabrication technology. This design alleviates the compressive effect of traditional rigid sensors on flap blood supply, demonstrating excellent stability and biocompatibility in 72-h continuous monitoring after free flap transplantation.^46^ However, contact-based technologies still suffer from inherent drawbacks such as susceptibility to local compression, potential infection risks, and interference from connecting wires, which limit their application in long-term dynamic monitoring.

### C. Infrared radiation temperature measurement technology

Infrared radiation temperature measurement technology, including infrared thermometers and infrared thermal imagers, realizes non-contact temperature measurement based on the principle of object thermal radiation and has become a mainstream technology in clinical flap monitoring. Infrared thermometers feature simple operation: they receive infrared radiation energy from the flap surface and convert it into temperature values, with a measurement time of only 1–2 s and an error controlled within ±0.5 °C.^47^ Its key advantage lies in its non-contact nature, which avoids mechanical stimulation and compressive effects of sensors on the flap. For fragile flap tissue in the early postoperative period, this technology not only ensures measurement safety but also minimizes the impact of external factors on flap blood supply, providing clinicians with true and reliable temperature reference data. Furthermore, dynamic and continuous monitoring of thermal images to track the evolution of the flap's temperature field can provide important evidence for predicting the progression of vascular crisis and evaluating the efficacy of treatment interventions. However, certain limitations persist in the clinical application of this technology: On the one hand, accurate interpretation of thermal images requires medical staff to receive professional training to distinguish between physiological temperature differences (e.g., the normal temperature gradient between the flap edge and center) and pathological temperature changes; on the other hand, environmental factors are prone to generating artifacts in thermal images, interfering with judgment accuracy. Based on this, infrared thermal imagers are currently mainly used in intensive flap monitoring in large medical centers, such as complex wound repair, flap monitoring after organ transplantation, and related clinical research fields.^48^

### D. Fiber optic sensor-based temperature measurement technology

The core principle of fiber optic sensor-based temperature measurement technology relies on using optical fibers as low-loss light-transmitting media. Temperature quantification is indirectly achieved by monitoring changes in phase, intensity, or wavelength characteristics of optical signals during transmission in fibers, which are induced by temperature variations. Endowed with advantages such as electromagnetic interference resistance and excellent chemical stability, this technology exhibits unique potential in the field of biomedical monitoring. Common temperature measurement mechanisms mainly include two types: the Bragg wavelength shift effect of fiber Bragg gratings (FBGs) and the fluorescence lifetime variation of rare earth-doped fibers.^49^ Among them, FBG sensors have emerged as a research focus in flap temperature monitoring due to their outstanding characteristics, including strong electromagnetic interference resistance, biocorrosion resistance, and miniaturized size.

FBG sensors function by writing a periodic refractive index modulation structure into the fiber core. When incident light meets the Bragg condition, reflected light of a specific wavelength is generated. Temperature changes alter the grating period and refractive index through thermal expansion/contraction effects and thermo-optic effects, resulting in a linear shift of the reflected wavelength.^50^ This characteristic enables their implantation into the subcutaneous tissue of the flap or fixation near the vascular pedicle, realizing multi-point distributed temperature measurement. Experimental studies have confirmed that FBG sensors can accurately capture temperature fluctuations at the 0.1 °C level in flap ischemia-reperfusion models, with a dynamic response time <1 s, providing high spatiotemporal resolution quantitative data for assessing the degree of metabolic damage. Furthermore, fiber optic sensing technology employs biocompatible materials, allowing for long-term *in vivo* implantation monitoring and effectively addressing the infection risk associated with traditional electronic sensors due to connecting wires. However, certain limitations remain in its clinical application: First, the high cost of FBG sensors and demodulation systems restricts large-scale popularization; Second, signal demodulation requires specialized spectral analysis equipment, leading to a high operational threshold; Third, macrobending loss is prone to occur when the fiber bending radius is less than 20 mm, causing signal attenuation and limiting its application in flap sites with high mobility such as peri-articular regions.^51^

### E. Microwave sensor-based temperature measurement technology

Microwave sensor-based temperature measurement technology achieves temperature sensing based on the characteristics of electromagnetic wave–tissue interaction. Its core principle lies in the significant temperature sensitivity of the dielectric constant of biological tissues—within the physiological temperature range, the tissue dielectric constant exhibits a linear variation with increasing temperature. This technology involves transmitting microwave signals of specific frequencies, receiving electromagnetic signals reflected or transmitted by tissues, and inverting the flap temperature distribution by extracting the characteristic changes of dielectric constant. It is classified as a prominent representative of non-contact deep temperature measurement technology.^52^ In preclinical studies on porcine free flap transplantation models, microwave temperature measurement technology has been proven effective in identifying deep tissue temperature decreases caused by vascular pedicle torsion. It detects abnormal signals 20–30 min earlier than surface infrared thermometry, achieving a sensitivity of 90% for the identification of early vascular crisis with a temperature measurement error controlled within ±0.6 °C.^53^ The underlying mechanism is that microwave signals are more sensitive to changes in water content and hemodynamics induced by altered tissue perfusion, enabling earlier capture of temperature fluctuations associated with metabolic abnormalities. However, the clinical application of this technology still faces several challenges: First, measurement accuracy is susceptible to interference from tissue composition—adipose tissue, with its low dielectric constant and weak temperature sensitivity, may lead to local measurement deviations, while high-water-content tissues (e.g., edematous flaps) can enhance electromagnetic wave attenuation and hinder signal extraction. These issues require optimization through multi-frequency fusion algorithms or tissue composition-adaptive correction models. Second, the integration level of current devices needs to be improved—mainstream systems are still desktop-based, weighing > 2 kg, and insufficient portability limits their bedside application. Third, signal analysis requires complex electromagnetic inverse problem solving algorithms, imposing high professional requirements on operators. Currently, microwave sensor-based temperature measurement technology is mainly in the stage of preclinical research and small-scale pilot applications, and its monitoring efficacy in flaps with complex tissue compositions (e.g., composite flaps containing bone and tendons) requires further verification.

Here, we present a comparative analysis of five primary temperature monitoring technologies for postoperative flap assessment. Table I summarizes the implementation principles, core advantages, and main limitations of each method, ranging from conventional digital palpation to advanced fiber optic and microwave sensing, providing a comprehensive overview for clinical and research applications.

**TABLE I.** Comparison of flap temperature monitoring technologies.

Technical category	Implementation principle	Core advantages	Main limitations	References
Finger palpation	Subjective tactile perception of temperature difference	Simple operation, no equipment required, low cost	Subjective, low accuracy, non-quantifiable, high miss rate	43
Contact temperature measurement	Temperature measurement through thermal conduction by direct contact of the sensor	High accuracy, fast response	Risk of compression, potential disruption of blood flow, not suitable for long-term continuous monitoring	44
Infrared radiation thermometer	Temperature values converted from the infrared radiation energy on the skin flap surface	Non-contact, safe and convenient, thermal imaging enables full-domain visualization	Susceptible to environmental interference, thermal image interpretation requires professional training, high cost	48
Fiber optic sensing	Utilizing the principle that temperature can change optical signals to achieve temperature measurement	High accuracy (0.1 °C level), anti-interference, can be implanted for long-term monitoring	High cost, complex system, high operation threshold, limited bending radius	49
Microwave sensing	Reflecting deep temperature through the emission/reception of microwaves based on the linear change of tissue dielectric constant with temperature	Deep temperature measurement, strong penetration, earlier warning	Susceptible to tissue composition interference, heavy equipment, complex algorithms	52

## V. APPLICATION OF INTELLIGENT TECHNOLOGIES IN POST-TRANSPLANTATION FLAP TEMPERATURE MONITORING

### A. Wearable temperature sensors

Endowed with the capability of continuous and real-time monitoring of flap temperature, wearable temperature sensing technology has emerged as a research focus in the field of post-transplantation flap monitoring in recent years. Fabricated with flexible electronic materials as the core substrate, such devices possess thin, lightweight, and flexible characteristics, enabling them to closely conform to the flap surface. Continuous collection of flap temperature data is achieved via built-in miniature temperature sensors; simultaneously, the devices are equipped with wireless transmission modules that can real-time transmit the collected data to terminal devices. This further realizes remote monitoring of flap temperature and abnormal alarm functions, providing convenient and efficient technical support for clinical monitoring.^54^ For instance, Bluetooth-enabled intelligent monitoring patches^55^ feature a compact size and lightweight design, causing minimal interference with patients' daily activities. They have been proven to enable continuous temperature monitoring with a measurement error within ±0.2 °C, which can meet the core clinical requirements for accurate flap temperature monitoring and have demonstrated excellent practicality in clinical trials. In terms of clinical application value, the core advantage of wearable temperature sensors is highlighted by their ability to dynamically capture the changing trend of flap temperature, overcoming the limitations of discontinuity associated with traditional intermittent monitoring and facilitating the early detection of flap vascular crisis by medical staff.^56^ Relevant multicenter study data have confirmed this advantage: patients using wearable temperature sensors had an average 2–3 h earlier detection of vascular crisis, and the flap survival rate was increased by 12% compared with traditional monitoring methods, securing valuable time for the early intervention of postoperative flap complications.

Despite the significant advantages of wearable temperature sensing technology, its current clinical promotion still faces three core challenges: First, the long-term adhesion stability between the sensor and the flap is susceptible to external factors—patients' sweat secretion, daily activities, and other factors may lead to sensor displacement or reduced adhesion, thereby causing unstable temperature data collection; Second, the anti-interference capability of wireless data transmission needs to be enhanced—in complex clinical environments, it is prone to being affected by equipment signals, environmental electromagnetic interference, etc., resulting in data transmission interruption or delay; Third, the regular calibration procedures for sensors lack unified standards—calibration methods vary greatly among devices of different brands and models, increasing the complexity of clinical operations. Currently, most wearable temperature sensors are in the clinical trial phase and have not yet achieved widespread popularization. In the future, through material innovation (e.g., adopting novel breathable and waterproof flexible materials) and algorithm optimization (e.g., implementing targeted noise reduction processing to improve data quality), wearable temperature sensing technology is expected to become the preferred option for post-transplantation flap monitoring.

### B. AI-driven dynamic temperature monitoring and early warning systems

The core principle of artificial intelligence (AI)-driven dynamic temperature monitoring and early warning systems lies in applying machine learning and deep learning algorithms for pattern recognition and feature extraction from continuous temporal temperature data, thereby establishing the mapping relationship between temperature changes and flap blood supply status and achieving abnormal early warning and risk prediction. Taking multi-source temperature monitoring data as input—including various types such as infrared thermal image sequences and fiber optic distributed temperature measurement data—the system captures the inherent regular features implied in temperature fluctuations through temporal neural networks [e.g., long short-term memory (LSTM) networks, transformer models] and integrates clinical prior knowledge to construct accurate early warning models.^57^

In recent years, numerous clinical studies have confirmed that AI-driven dynamic temperature monitoring and early warning systems can achieve early and efficient identification of postoperative risks such as flap venous crisis. For instance, the LSTM model trained on postoperative temperature data of 1200 free flaps achieved a prediction accuracy of up to 92.3% for arterial crisis, and the early warning time was 4.2 ± 1.5 h earlier than that of traditional manual monitoring, securing critical time for clinical intervention;^58^ furthermore, the multi-feature XGBoost algorithm model integrating temperature data and clinical signs improved the sensitivity of venous crisis diagnosis to 94.1%, significantly reducing the risk of missed diagnosis and further ensuring the effectiveness of postoperative flap monitoring.^59^ In terms of technical advantages, the system overcomes the limitations of traditional manual monitoring: On the one hand, it avoids the subjectivity of manual monitoring; On the other hand, it addresses the lag of manual monitoring. Through automated algorithmic analysis, the system enables a closed-loop management process encompassing “temperature abnormality identification—risk classification assessment—clinical intervention recommendation,” making postoperative flap monitoring more efficient and accurate. However, the current clinical promotion of this technology still faces three significant limitations: First, the model training data are mostly derived from single medical centers, leading to sample distribution bias. This may result in insufficient generalization ability of the model across different medical scenarios, affecting its applicability in extensive clinical practice; Second, the internal logic of complex algorithms has weak interpretability, making it difficult for clinical medical staff to clearly understand the generation basis of early warning results, which, to a certain extent, reduces their trustworthiness in the technology; Third, algorithm performance is highly dependent on high-quality annotated data, but the sample size of postoperative flap adverse events such as vascular crisis is relatively limited. This could lead to decreased prediction stability of the model in rare risk scenarios.

To address the aforementioned issues, the future clinical promotion of this technology needs to focus on two key directions: First, integrating explainable AI (XAI) technologies to optimize model transparency, making the generation logic of early warning results more comprehensible to medical staff and enhancing the clinical acceptability of the technology; Second, conducting multicenter collaboration to expand the scope of data collection, enrich sample types, reduce data bias, further improve the model's generalization ability and clinical applicability, and promote the widespread application of AI-driven dynamic temperature monitoring and early warning systems in the field of postoperative flap monitoring.

### C. Implantable flap monitoring sensors driven by flexible electronics technology

Adopting biomechanical compatibility as the core design philosophy, this technology achieves minimally invasive implantation into flap tissue through the synergistic optimization of flexible substrate materials and stretchable circuit structures. This design effectively avoids mechanical stimulation-induced tissue interference while enabling stable acquisition of flap temperature signals. Its core innovative value lies in the achievement of in-depth synergistic design of material–structure–function.^60^ With the continuous iteration of technology, such sensors have broken through the limitations of traditional single temperature monitoring and successfully achieved multi-dimensional functional expansion, providing a more comprehensive quantitative basis for the accurate assessment of flap blood supply status and metabolic level. Existing studies have reported that flexible thin-film sensors have realized temperature-pressure dual-parameter collaborative monitoring. Through the coupled analysis of temperature gradient and pressure distribution, abnormal conditions such as hematoma compression can be accurately identified, offering timely early warning for clinical intervention; furthermore, implantable fiber-optic-flexible composite sensors possess the capability of simultaneous monitoring of deep flap temperature and tissue oxygen partial pressure, which further enriches the dimensions of metabolic status assessment and provides more three-dimensional data support for medical research and clinical practice. At the animal experiment level, hydrogel-based sensors have demonstrated excellent stability in porcine flap transplantation models, enabling continuous operation for up to 28 days, which fully verifies their good biocompatibility.

Currently, the large-scale promotion of this technology still faces bottlenecks, centered on miniaturization breakthroughs in wireless power supply and signal transmission: First, although power supply modules based on near-field communication (NFC) technology have achieved a miniaturized volume of 1 cm^3^, the signal transmission distance is limited to within 5 cm, failing to meet the clinical needs of large-area flap monitoring; Second, energy harvesting technologies represented by body temperature thermoelectric power generation currently have an output power of less than 10 *μ*W, which cannot support the long-term stable operation of complex sensing modules. The aforementioned issues urgently require further breakthroughs through approaches such as material modification and upgrading as well as circuit structure optimization.

### D. Multimodal fusion monitoring technology

The principle of multimodal fusion monitoring technology is based on the physiological correlation of “temperature-blood flow-metabolism.” By integrating temperature signals with other key physiological parameters (e.g., blood perfusion, oxygen saturation, microcirculation images), a comprehensive model for flap blood supply assessment is constructed using multi-source data fusion algorithms.^61^ In recent years, multimodal systems have achieved hardware integration and algorithm optimization. At the hardware level, integrated integration of devices with different monitoring modalities has been realized. For instance, the integrated system of infrared thermal imagers and laser Doppler flowmeters has improved the prediction accuracy of flap necrosis to 89.7% through feature-level fusion algorithms, representing a 15.3% increase compared with the single infrared modality;^62^ the three-modal system combining temperature, oxygen saturation, and microcirculation images has achieved a detection sensitivity of 96.2% for early venous congestion in rabbit ear flap models. In terms of algorithms, decision-level fusion algorithms have become the core technology to address the issue of multimodal data heterogeneity.

The adoption of algorithms such as Dempster-Shafer (D-S) evidence theory and Bayesian networks can effectively address the spatiotemporal asynchrony of multimodal data, reduce misjudgments caused by fluctuations in a single parameter, and improve the reliability of assessment results. The main challenges currently facing this technology focus on three aspects: (1) Equipment integration is constrained by size and weight—existing multimodal devices fail to meet bedside portability requirements due to the integration of multiple sensing modules; (2) Stringent requirements are imposed on synchronous multi-source data acquisition: the temporal error of data from different modalities must be controlled within 10 ms, otherwise the dynamic correlation of physiological parameters will be undermined; (3) The anti-interference capability in clinical settings needs to be improved—factors such as strong light from surgical lamps, patient body position changes, and electromagnetic radiation from electrocardiographic monitoring equipment are prone to causing signal distortion in some modalities. Currently, this technology is in a critical stage of translation from laboratory research to clinical application. The core breakthrough directions in the future will focus on two aspects: first, achieving device miniaturization through miniaturized sensing chips and lightweight structural design; second, developing adaptive anti-interference algorithms to improve data acquisition quality in complex clinical environments, thereby promoting the clinical implementation and application of the technology.

Furthermore, intelligentization constitutes the core development direction in the future, with a focus on the in-depth integration of artificial intelligence (AI) and machine learning technologies with temperature monitoring. By training prediction models based on temperature change curves, early warning of vascular crisis and risk stratification will be realized. Meanwhile, intelligent decision support systems can be developed to automatically recommend intervention strategies based on temperature data and individual patient characteristics, assisting in clinical decision-making. This is particularly tailored to primary hospitals and young medical professionals.

Integrated development will promote the integration of monitoring technologies with therapeutic functions, facilitating the development of integrated devices featuring both temperature monitoring and local therapeutic capabilities. For example, integrating temperature control modules into wearable sensors allows for automatic activation of local heating to prevent vasospasm when abnormally low flap temperatures are detected; alternatively, incorporating drug delivery systems can release vasodilators or antibiotics in response to temperature abnormalities, enabling precision therapy.

Additionally, multidisciplinary collaboration serves as the key to technological breakthroughs, requiring collaborative innovation among experts in fields such as medicine, materials science, electronic engineering, and computer science. For instance, materials scientists develop sensor materials with enhanced flexibility, breathability, and biocompatibility; electronic engineers optimize wireless transmission technologies and low-power consumption designs; computer scientists develop efficient data analysis algorithms; and clinicians provide clinical requirements and validation feedback. Through interdisciplinary cooperation, it is expected to overcome existing technical bottlenecks and develop more accurate, convenient, and intelligent flap temperature monitoring technologies, thereby making greater contributions to improving the success rate of flap transplantation and enhancing patients' quality of life.

## VI. CONCLUSION

The development of flap transplantation temperature monitoring technology is undergoing a paradigm shift—from “single-parameter monitoring” to “multi-parameter fusion,” from “manual judgment” to “intelligent early warning,” and from “laboratory research” to “clinical application.” While significant progress has been achieved in this field, it is crucial to acknowledge the limitations of existing research. First, potential publication bias may exist in the included literature, as studies reporting positive outcomes of temperature monitoring technologies are more likely to be published than those with negative or inconclusive results, which may overestimate the actual efficacy of the technologies. Second, many emerging temperature monitoring technologies, such as wearable flexible sensors and intelligent early warning systems, lack long-term follow-up data; most current studies focus on short-term performance and safety, while the long-term stability, durability, and long-term impact on flap survival and patient prognosis remain unclear. Additionally, the current research on temperature monitoring technologies is mostly single-center, small-sample studies, and the lack of large-scale, multicenter randomized controlled trials limits the generalizability of the research conclusions.

In the future, with the advancement of precise monitoring systems, the widespread application of intelligent technologies, and the improvement of standardized clinical pathways, temperature monitoring will truly evolve into the core pillar of “precision early warning and timely intervention” in flap transplantation. This will provide a solid guarantee for boosting the success rate of flap transplantation and improving patient prognosis.

## Data Availability

The data that support the findings of this study are available from the corresponding authors upon reasonable request.
